# Matrine Suppresses Arsenic-Induced Malignant Transformation of SV-HUC-1 Cells via NOX2

**DOI:** 10.3390/ijms25168878

**Published:** 2024-08-15

**Authors:** Lanfei Wang, Nianfeng Qiu, Suyuan Tong, Yan Yu, Shuhua Xi, Fei Wang

**Affiliations:** 1 Key Laboratory of Environmental Stress and Chronic Disease Control and Prevention, Ministry of Education, China Medical University, Shenyang 110122, China; abhge77@gmail.com (L.W.); 17740080383@163.com (N.Q.); 18844227767@163.com (S.T.); yy1686527@163.com (Y.Y.); shxi@cmu.edu.cn (S.X.); 2 Department of Environmental Health, School of Public Health, China Medical University, Shenyang 110122, China; 3 Key Laboratory of Liaoning Province on Toxic and Biological Effects of Arsenic, China Medical University, Shenyang 110122, China

**Keywords:** arsenic, bladder cancer, matrine, NADPH Oxidase 2, NLRP3

## Abstract

Arsenic (As) has been classified as a carcinogen for humans. There is abundant evidence indicating that arsenic increases the risk of bladder cancer among human populations. However, the underlying mechanisms have yet to be fully understood and elucidated. NADPH oxidases (NOXs) are the main enzymes for ROS production in the body. NADPH Oxidase 2 (NOX2), which is the most distinctive and ubiquitously expressed subunit of NOXs, can promote the formation and development of tumors. The utilization of NOX2 as a therapeutic target has been proposed to modulate diseases resulting from the activation of NOD-like receptor thermal protein domain associated protein 3 (NLRP3). Matrine has been reported to exhibit various pharmacological effects, including anti-inflammatory, antifibrotic, antitumor, and analgesic properties. However, it has not been reported whether matrine can inhibit malignant transformation induced by arsenic in uroepithelial cells through NOX2. We have conducted a series of experiments using both a sub-chronic NaAsO_2_ exposure rat model and a long-term NaAsO_2_ exposure cell model. Our findings indicate that arsenic significantly increases cell proliferation, migration, and angiogenesis in vivo and in vitro. Arsenic exposure resulted in an upregulation of reactive oxygen species (ROS), NOX2, and NLRP3 inflammasome expression. Remarkably, both in vivo and in vitro, the administration of matrine demonstrated a significant improvement in the detrimental impact of arsenic on bladder epithelial cells. This was evidenced by the downregulation of proliferation, migration, and angiogenesis, as well as the expression of the NOX2 and NLRP3 inflammasomes. Collectively, these findings indicate that matrine possesses the ability to reduce NOX2 levels and inhibit the transformation of bladder epithelial cells.

## 1. Introduction

Arsenic is a metalloid element widely existing in nature, particularly present at high levels in the groundwater of many countries [1]. The World Health Organization reported that about 140 million people in 70 countries are at risk of arsenic exposure in drinking water. This has created an important public health issue around the world [2]. It can enter the body, causing damage to multiple organs [3]. The International Agency for Research on Cancer (IARC) has classified arsenic as a Group 1 carcinogen for humans [4]. Chronic exposure to arsenic can lead to diabetes, cardiovascular, and neurological disorders and is associated with different types of cancer, including skin [5], lung [6], and bladder cancers [7].

Several studies have examined various characteristics that may be associated with the carcinogenic effects of arsenic-related bladder cancer. About 70–90% of inorganic arsenic (iAs^3+^ and iAs^5+^) is absorbed by the gastrointestinal tract, then distributed through the bloodstream to different organs, predominantly the liver, kidneys, lungs, and bladder, and secondarily to muscle and nerve tissue [8]. The major effects of arsenic in the urinary tract are kidney and bladder cancer. Bladder cancer is the ninth most common cancer worldwide [4]. Ingested inorganic arsenic is metabolized by methylation through a detoxification pathway. Arsenic methylation capacity can be assessed based on urinary levels of arsenic metabolites, including arsenite, arsenate, monomethyl arsenic acid, and dimethyl arsenic acid, which are reliable markers of cumulative arsenic exposure [9]. Although there are many tumor markers available to identify bladder cancer [10], the underlying mechanism of arsenic-induced bladder cancer is unclear now. Arsenic-induced bladder cancer continues to show high rates around the world. Bladder cancer mainly occurs in the epithelial cell layer, and the SV-HUC-1 cell line is a type of immortalized normal bladder epithelial cell line. Our group has found that arsenic exposure can induce abnormal proliferation of bladder epithelial cells in vivo and the malignant behavior of human ureteral epithelial (SV-HUC-1) cells [11,12].

NADPH oxidases (NOXs) are a class of multi-subunit transmembrane enzymes that utilize reduced nicotinamide adenine dinucleotide phosphate (NADPH) as an electron donor to catalyze the reduction of oxygen into superoxide anion (O_2_^•−^) and hydrogen peroxide (H_2_O_2_). Initially discovered in neutrophils and macrophages during the inflammatory response, NOXs serve as the primary enzymes accountable for the generation of reactive oxygen species (ROS) within the human body [13]. ROS acts as a signaling molecule that mediates cell proliferation, migration, and angiogenic gene expression in cancer cells. ROS, derived from NADPH oxidases, plays a significant role in the physiology and pathology of cancer [14].

NADPH Oxidase 2 (NOX2) is the most distinctive and widely distributed subunit of NADPH oxidases, mainly in the thymus, small intestine, colon, spleen, pancreas, ovary, placenta, prostate, and testis [13]. NOX2 can limit inflammation and injury by modulating key signaling pathways that affect neutrophil accumulation and clearance [15]. ROS generated by NOX2 can also promote the formation and development of tumors through an immunological pathway [16]. NOX2 expression in ESCC cells affects tumorigenesis, especially cell cycle progression [17]. In the study of prostate cancer, it was found that NOX2 can further control the development of the tumor vascular system and limit the development and metastasis of prostate tumors by regulating Vascular endothelial-derived growth factor (VEGF) signaling [18]. NOX2, therefore, may be a key target for the treatment of tumors.

The NLRP3 (NOD-like receptor thermal protein domain associated protein 3) inflammasome plays a crucial role in the innate immune system. It is responsible for activating Caspase 1 and triggering the release of proinflammatory cytokines IL-1β and IL-18 in response to microbial infections and cellular damage [19]. NLRP3 has been directly implicated in the pathogenesis of various inflammatory diseases and exhibits a dual function in the development of metabolic disorders and cancer [20]. However, it should be noted that the significance of NLRP3 as a potential marker in tumor therapy cannot be overlooked. Genetic variations in the gene encoding the NLRP3 inflammasome pathway have been found to be linked to the occurrence of malignant tumors, including chronic myeloid leukemia [21] and melanoma [22]. A study has identified the involvement of NOX2 in the induction of oxidative stress during the pathological progression of Traumatic Brain Injury (TBI). Knocking out NOX2 leads to a decrease in the activation of the NLRP3 inflammasome and the expression of inflammatory components [23]. This finding implies that targeting NOX2 may have the potential to regulate diseases that arise from NLRP3 activation.

For a long time, natural products have been considered the key potential drugs to treat a series of fatal diseases, including cancer. A variety of natural products isolated from Chinese herbs are extensively employed in cancer treatment for their excellent antiproliferation, pro-apoptosis, anti-metastasis, autophagy, and immune regulation activities [24]. Matrine (C_15_H_24_N_2_O) is one of the key tetracyclo-quinolizindine alkaloids isolated from the roots of Sophora flavescens (Kushen) [25]. It has been reported to possess anti-inflammatory, antifibrotic [26], antitumor, and analgesic pharmacological effects [27]. A study has shown that matrine can significantly reduce oAβ (oligomeric Aβ)-induced protein expression of NADPH oxidase [28]. Studies have also demonstrated that matrine can suppress colorectal cancer cell proliferation both in vitro and in vivo [29]. Additionally, it has been found to suppress lung cancer metastasis by targeting M2-like tumor-associated macrophage polarization [30]. Matrine can block the PTEN/PI3K/AKT pathway in bladder cancer cells through the upregulation of LINC00472/pdcd4 [31]. Furthermore, it can inhibit the migration and invasion of pancreatic cancer cells PANC-1 through the ROS/NF-κB/MMPs pathway [32]. However, it is unclear whether matrine can inhibit arsenic-induced bladder epithelial cell proliferation (such as CyclinD1, Proliferating Cell Nuclear Antigen (PCNA)), migration (such as E-cadherin and vimentin), and angiogenesis (such as VEGF) through NOX2.

ROS and inflammatory response are closely related to tumorigenesis and development, and inhibition of NOX2 can limit the occurrence of inflammation and the generation of ROS, but there is no literature report on inhibiting NOX2 to delay the occurrence and development of arsenic-induced bladder cancer. We established a rat model of subchronic inorganic arsenic exposure and an in vitro model of bladder epithelial cells with long-term low-dose arsenic exposure to investigate whether NOX2 can regulate arsenic-induced bladder epithelial cell proliferation, migration, and angiogenesis through ROS and inflammatory responses, as well as the intervention effect of natural plant matrine on NOX2 and the alleviation of malignant transformation. Generally, our study is to find out whether the action of matrine in SV-HUC-1 cells via the NOX2 pathway can provide a potential therapeutic strategy for arsenite-induced bladder cancer.

## 2. Results

### 2.1. Effect of Long-Term Arsenite Exposure on Proliferation in SV-HUC-1 Cells

**Figure 1:**
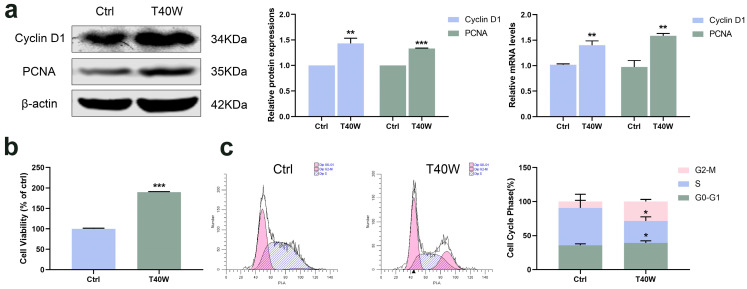
Effect of long-term arsenite exposure in SV-HUC-1 cells. (**a**) The protein and mRNA expressions of Cyclin D1 and PCNA. (**b**) Comparison of cell viability between the Ctrl and T40W groups. (**c**) Representative flow histograms and cell cycle distribution in Ctrl and T40W groups. Data were presented as mean ± SD. * *p* < 0.05, ** *p* < 0.01, and *** *p* < 0.001 compared to the Ctrl cells (n = 3). Ctrl: SV-HUC-1 cells; T40W: SV–HUC–1 cells treated with 0.5 μM arsenite for 40 weeks.

Cyclin D1 has been identified as a proto-oncogene, and its overexpression can cause uncontrolled cell proliferation and malignancy [33]. PCNA is a polypeptide chain that is considered a molecular marker for proliferation. Long-term arsenite exposure increased the protein and mRNA levels of Cyclin D1 and PCNA compared to the untreated SV-HUC-1 cells (Figure 1a). And cell viability significantly increased with the long-term arsenite treatment (Figure 1b). At the same time, arsenite treatment decreased the proportion of cells in the S phase and increased that in the G2 phase, thus accelerating cell cycle progress (Figure 1c). It can be seen that, therefore, arsenite may induce hyperproliferation of bladder epithelial cells by speeding up the cell cycle progression.

### 2.2. Effect of Long-Term Arsenite Exposure on Migration and Angiogenesis in SV-HUC-1 Cells

**Figure 2:**
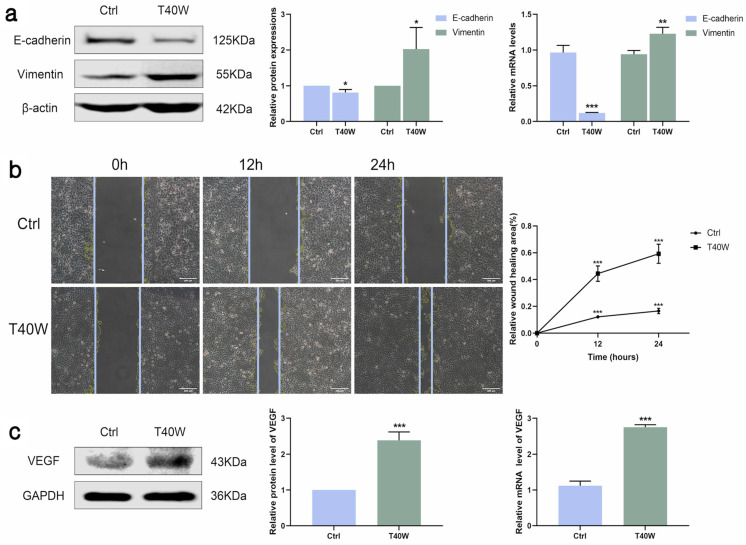
Effect of long-term arsenite exposure on migration and angiogenesis in SV-HUC-1 cells. (**a**) The protein and mRNA expressions of E–cadherin and Vimentin. (**b**) Representative images of cell proliferation and migration examined by wound healing assay between Ctrl and T40W groups, bar = 200 μm. (**c**) The protein and mRNA expressions of VEGF. Data were presented as mean ± SD. * *p* < 0.05, ** *p* < 0.01, and *** *p* < 0.001 compared to the Ctrl cells (n = 3). Ctrl: SV-HUC-1 cells; T40W: SV-HUC-1 cells treated with 0.5 μM arsenite for 40 weeks.

E-cadherin and vimentin are epithelial and mesenchymal cell markers. The shortage of E-cadherin expression and upregulation of vimentin are the most important characteristics of epithelial-to-mesenchymal transition (EMT). As shown in Figure 2a, long-term arsenite treatment downregulated the protein and mRNA expressions of E-cadherin and upregulated vimentin. Furthermore, as time goes by, long-term arsenite exposure increases the migration of SV-HUC-1 cells (Figure 2b).

Angiogenesis is indispensable for the occurrence and development of tumors. VEGF is a key factor in angiogenesis and fosters EMT in the hypoxic microenvironment. Arsenite exposure significantly increased the protein and mRNA expressions of VEGF (Figure 2c). Taken together, long-term arsenite exposure can promote proliferation, migration, and angiogenesis in SV-HUC-1 cells.

### 2.3. Chronic Arsenite Treatment Activates the Expression of NLRP3 Inflammasome and NOX2 in SV-HUC-1 Cells

**Figure 3:**
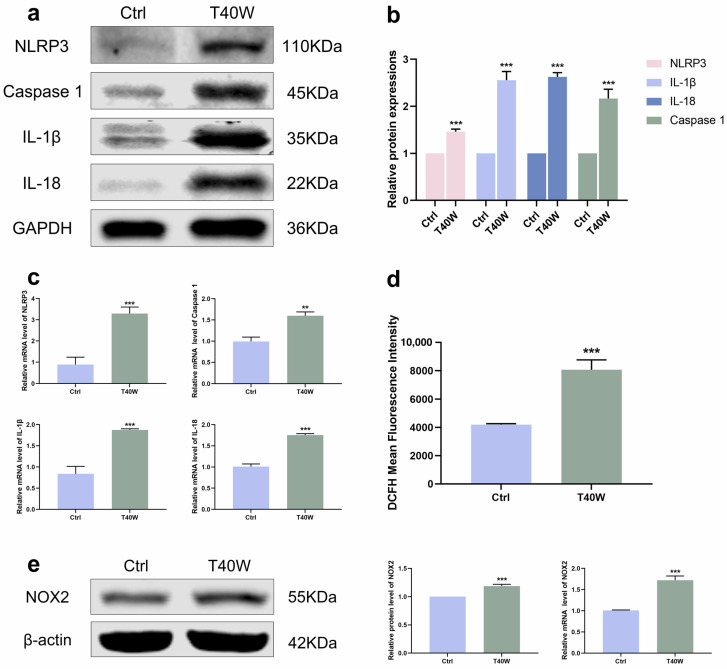
Arsenite exposure increased the expressions of NLRP3 inflammasome and NOX2. (**a**–**c**) The protein and mRNA expressions of NLRP3, Caspase 1, IL−1β, and IL−18. (**d**) ROS generation was measured using DCFH−DA by flow cytometry. (**e**) The protein and mRNA expressions of NOX2. Data were presented as mean ± SD. ** *p* < 0.01 and *** *p* < 0.001 compared to the Ctrl cells (n = 3). CASP1: Caspase 1; Ctrl: SV-HUC-1 cells; T40W: SV-HUC-1 cells treated with 0.5 μM arsenite for 40 weeks.

The development of tumors has a close relationship with inflammation. According to Figure 3a–c, the NLRP3 inflammasome and downstream factors (Caspase 1, IL−1β and IL−18) showed an upward trend under the exposure of chronic arsenite treatment in protein and mRNA levels. NOXs is the membrane-bound enzyme responsible for the generation of reactive oxygen species (ROS). With exposure to arsenite, the generation of ROS increased (Figure 3d). Consistent with this, the protein and mRNA levels of NOX2 were also significantly increased by chronic arsenite treatment (Figure 3e). Gene silencing of NOX2 can inhibit OGD-induced NLRP3 expression [34]. Summing up the results, arsenite can induce hyperexpression of NLRP3 and NOX2. But whether NOX2 can mediate arsenic-induced NLRP3 expression is unclear now.

### 2.4. Arsenite-Induced Overexpression of NLRP3 Inflammasome and NOX2 Decreased by Matrine In Vitro

**Figure 4:**
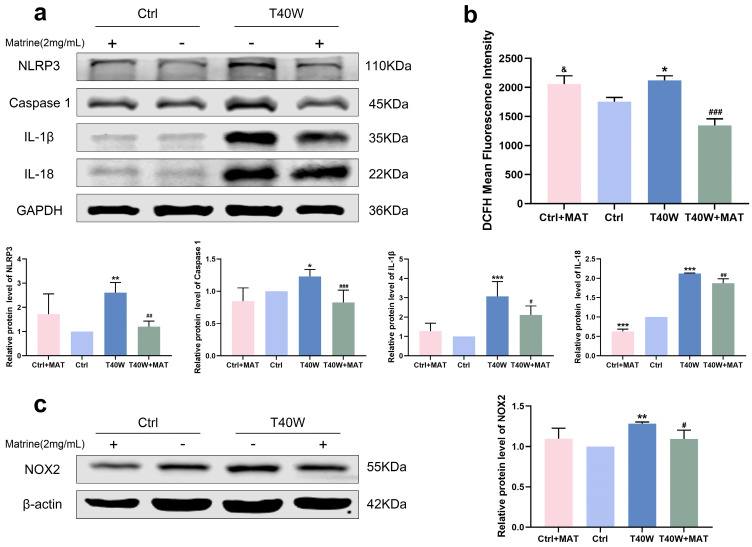
Matrine decreased overexpression of NLRP3 inflammasome and NOX2 in vitro. (**a**) Expression of NLRP3, Caspase 1, IL−1β, and IL−18 proteins in cells treated with matrine. (**b**) ROS generation in cells treated with matrine. (**c**) Expression of NOX2 protein in cells treated with matrine. Data were presented as mean ± SD. * *p* < 0.05, ** *p* < 0.01, and *** *p* < 0.001 compared to the Ctrl cells (n = 3). # *p* < 0.05, ## *p* < 0.01, and ### *p* < 0.001 compared to the T40W cells (n = 3). & *p* < 0.05 compared to T40W+MAT. MAT: matrine; Ctrl: SV-HUC-1 cells; T40W: SV-HUC-1 cells treated with 0.5 μM arsenite for 40 weeks.

For a long time, natural products have been considered the key potential drugs to treat a series of fatal diseases, including cancer. A study shows that matrine inhibits tumor growth, invasion, and migration in vivo by upregulating LINC00472/pdcd4 to inhibit the PTEN/PI3K/AKT pathway of bladder cancer cells [31]. To explore the role of matrine in arsenite-treated SV-HUC-1 cells, we diluted it to 2 mg/mL for the following experiments. We discovered that matrine can attenuate the high protein expression levels of the NLRP3 inflammasome through chronic arsenite treatment in SV-HUC-1 cells (Figure 4a). We found that matrine precluded arsenite-induced ROS hoisting (Figure 4b). Furthermore, in SV-HUC-1 cells, matrine inverts the overexpression of NOX2 by arsenic treatment (Figure 4c). It could be a clue to discovering the relationship between NLRP3 and NOX2.

### 2.5. Matrine Suppressed the Expression of NLRP3 Inflammasome via Inhibiting NOX2 In Vivo

**Figure 5:**
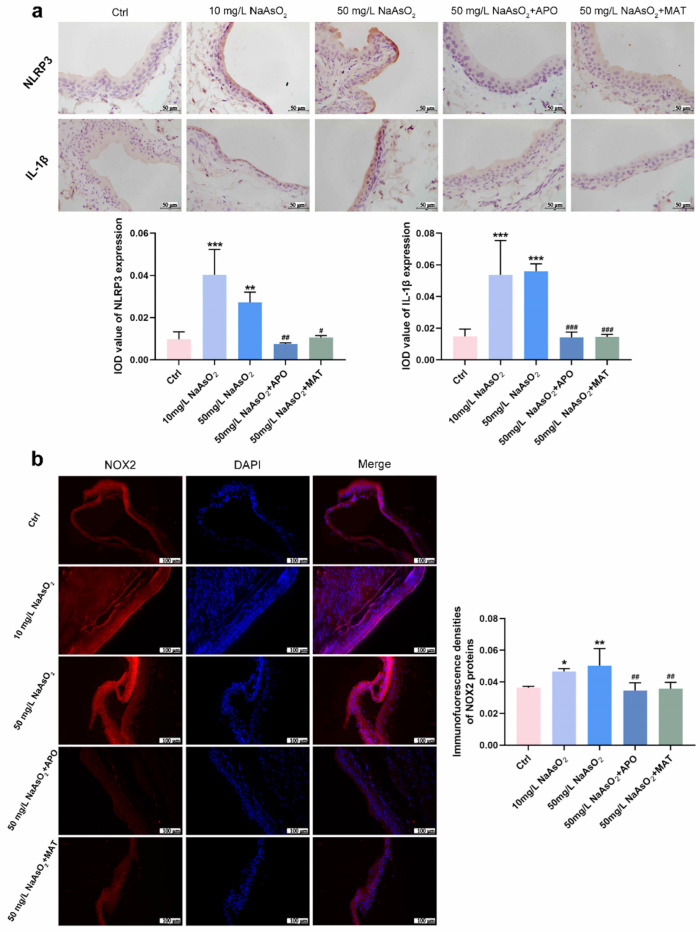
Matrine suppressed the expression of NLRP3 inflammasome by inhibiting NOX2 in vivo. (**a**) Immunohistochemical staining of NLRP3 and IL-1β in the bladder epithelia of rats treated with arsenite and/or apocynin/matrine (400×), bar = 50 μm. (**b**) Immunofluorescence staining of NOX2 in the bladder epithelia of rats treated with arsenite and/or apocynin/matrine (200×), Bar = 100 μm. Data were presented as the mean ± SD of 4 rats. * *p* < 0.05, ** *p* < 0.01, and *** *p* < 0.001 compared to the Ctrl group. # *p* < 0.05, ## *p* < 0.01, and ### *p* < 0.001 compared to the 50 mg/L NaAsO_2_ group. APO: apocynin treated; MAT: matrine treated.

To explore whether the reduction of the NLRP3 inflammasome was regulated by matrine via the NOX2 pathway, we treated it with apocynin (a NOX2 inhibitor) and matrine in vivo. Thirty Wistar rats (5-week-old) were randomly divided into 5 groups according to rat weight: Control group (drinking distilled water for 12 weeks); 10 mg/L NaAsO_2_ group (drinking 10 mg/L NaAsO_2_ in distilled water freely for 12 weeks); 50 mg/L NaAsO_2_ group (drinking 50 mg/L NaAsO_2_ in distilled water freely for 12 weeks); 50 mg/L NaAsO_2_ + APO group (drinking 50 mg/L NaAsO_2_ in distilled water freely for 12 weeks and giving 20 mg/kg apocynin (APO) daily by gavage starting from the 9th week); 50 mg/L NaAsO_2_ + MAT group (drinking 50 mg/L NaAsO_2_ in distilled water freely for 12 weeks and giving 20 mg/kg matrine (MAT) daily by gavage starting from the 9th week). Immunostaining shown in Figure 5a found that NLRP3 and IL-1β levels were significantly upregulated in the bladder epithelial cells of the 10 mg/L and 50 mg/L arsenite-treated groups compared to the control group and obviously decreased following apocynin and matrine treatment. In keeping with the results, NOX2 levels were markedly upregulated in the bladder epithelial cells of arsenite-exposed rats compared to the control group, and decreased significantly with apocynin and matrine interruption (Figure 5b). In summary, matrine may suppress the expression of the NLRP3 inflammasome by inhibiting NOX2.

### 2.6. Matrine Decreased the Proliferation and Cell Viability in Bladder Epithelial Cells

**Figure 6:**
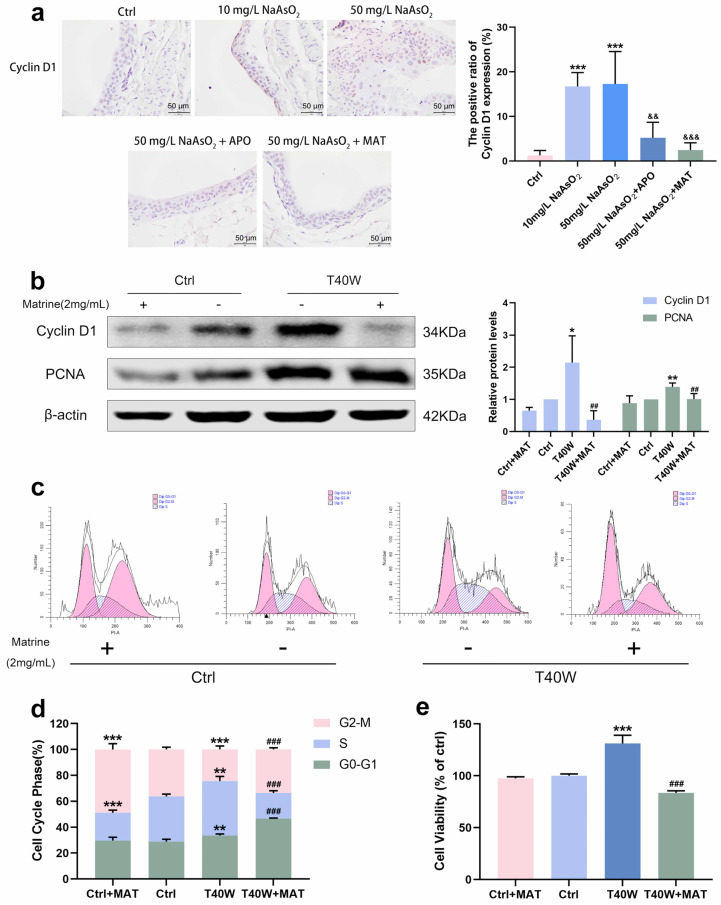
Matrine decreased the proliferation and cell viability of bladder epithelial cells. (**a**) Immunohistochemical staining of Cyclin D1 in the bladder epithelia of rats treated with arsenite and/or apocynin/matrine (400×), Bar = 50 μm. Data were presented as mean ± SD. *** *p* < 0.001 compared to the Ctrl group. && *p* < 0.01 and &&& *p* < 0.001 compared to the 50 mg/L NaAsO_2_ group. (**b**) Expression of Cyclin D1 and PCNA proteins in SV−HUC-1 cells treated with matrine. (**c**,**d**) Representative flow histograms and cell cycle distribution in the Ctrl, Ctrl + MAT, T40W, and T40W + MAT groups of SV-HUC-1 cells. (**e**) Cell viability of Ctrl, Ctrl + MAT, T40W, and T40W + MAT groups of SV-HUC- cells. Data were presented as mean ± SD. * *p* < 0.05, ** *p* < 0.01, and *** *p* < 0.001 compared to the Ctrl group. ## *p* < 0.01 and ### *p* < 0.001 compared to the T40W cells (n = 3). APO: apocynin-treated; MAT: matrine-treated; T40W: SV-HUC-1 cells treated with 0.5 μM arsenite for 40 weeks.

As matrine can inhibit NLRP3 expression in bladder epithelial cells via NOX2, we wonder what role matrine will play in arsenic-induced hyperproliferation. Immunohistochemical analysis of rats in the arsenite-treated groups showed the presence of CyclinD1 brown DAB positive cells sensibly, whereas few positive cells were detected in the apocynin and matrine groups (Figure 6a). In SV-HUC-1 cells, the presentation of Cyclin D1 and PCNA protein levels decreased with the treatment of matrine compared to the T40W SV-HUC-1 cells (Figure 6b). Cell cycle analysis shows that matrine reduced the proportion of cells in the S phase and increased that in the G2-M phase and G0-G1 phase compared to the T40W cells (Figure 6c,d). It indicated that matrine could slow down the progress of the cell cycle caused by arsenite. Plus, matrine significantly mitigated the cell viability in T40W cells (Figure 6e). The results suggest that matrine may reverse arsenite-induced cell proliferation through the NOX2 pathway.

### 2.7. Matrine Regulated E-Cadherin and Vimentin and Diminished the Ability of Migration in Bladder Epithelial Cells

**Figure 7:**
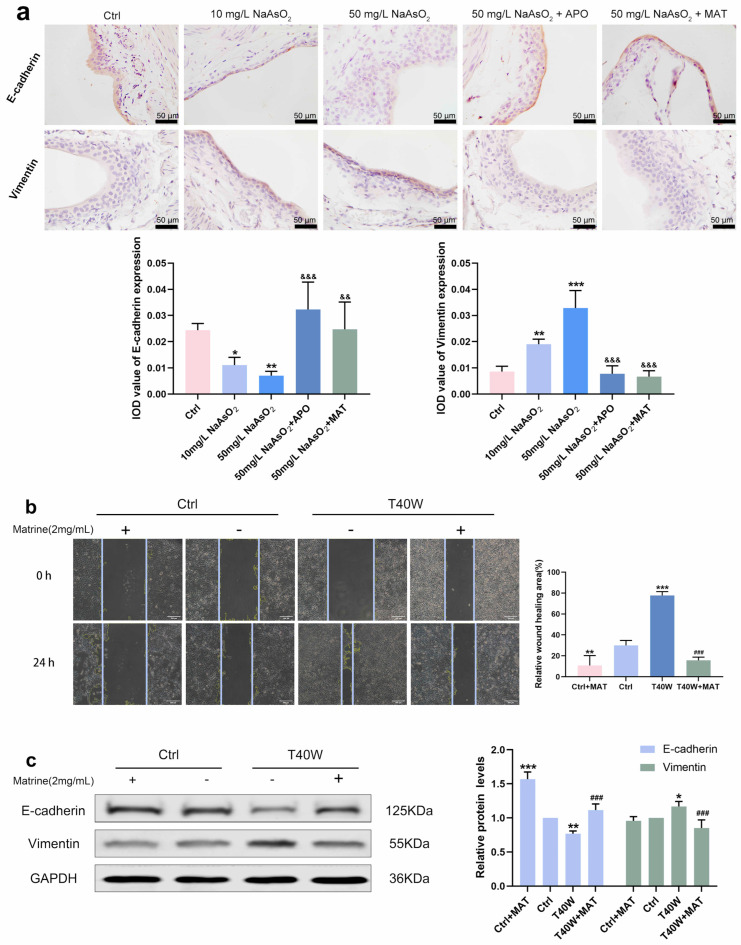
Matrine regulated E-cadherin and Vimentin and diminished the ability to migrate in bladder epithelial cells. (**a**) Immunohistochemical staining of E-cadherin and Vimentin in the bladder epithelia of rats treated with arsenite and/or apocynin/matrine (400×), Bar = 50 μm. (**b**) Representative images of cell proliferation and migration examined by wound healing assay, bar = 200 μm. (**c**) Expression of E–cadherin and Vimentin proteins in cells treated with matrine. Data were presented as mean ± SD. * *p* < 0.05, ** *p* < 0.01, and *** *p* < 0.001 compared to the Ctrl group. ### *p* < 0.001 compared to the T40W cells (n = 3). && *p* < 0.01 and &&& *p* < 0.001 compared to the 50 mg/L NaAsO_2_ group. APO: apocynin-treated; MAT: matrine-treated; T40W: SV-HUC-1 cells treated with 0.5 μM arsenite for 40 weeks.

Immunostaining intensity of E–cadherin in arsenite-treated groups increased, and Vimentin decreased compared to the control group, but it was all inversed by apocynin and matrine in vivo (Figure 7a). As for the migration of the cells, the wound of T40W became narrow after 24 h. However, matrine broadens the wound significantly (Figure 7b). Protein changes in E-cadherin and Vimentin in vitro are the same as the immunohistochemical changes (Figure 7c). The results reveal that the conversions of E-cadherin, Vimentin, and migration in bladder epithelial cells are regulated by matrine through NOX2.

### 2.8. Matrine Declined the Angiogenesis in Bladder Epithelial Cells

**Figure 8:**
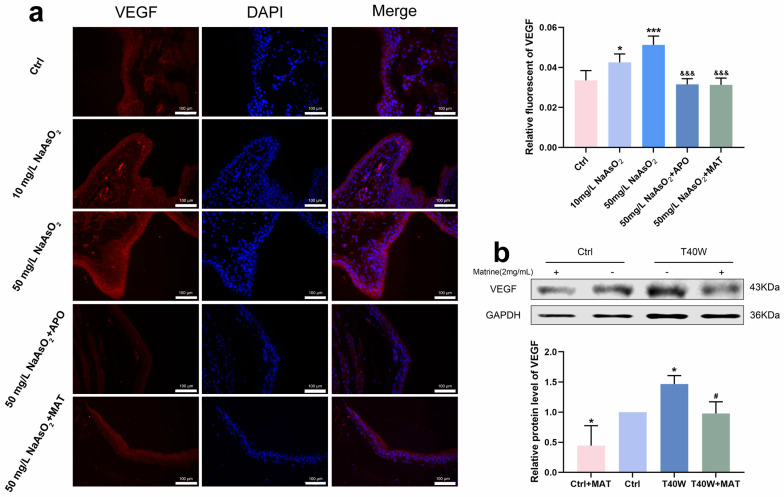
Matrine declined the angiogenesis in bladder epithelial cells. (**a**) Immunofluorescence staining of VEGF in the bladder epithelia of rats treated with arsenite and/or apocynin/matrine (200×), bar = 100 μm. (**b**) Expression of VEGF protein in cells treated with matrine. Data were presented as mean ± SD. * *p* < 0.05 and *** *p* < 0.001 compared to the Ctrl group. # *p* < 0.05 compared to the T40W cells (n = 3). &&& *p* < 0.001 compared to the 50 mg/L NaAsO_2_ group. APO: apocynin-treated; MAT: matrine-treated; T40W: SV-HUC-1 cells treated with 0.5 μM arsenite for 40 weeks.

According to Figure 8a, it had a high fluorescence intensity in the 10 mg/L and 50 mg/L groups, which promoted the overexpression of VEGF. It has shown the downregulation of VEGF in rats’ bladder epithelial cells through the interruption of apocynin and matrine. Moreover, matrine also decreased the protein level of VEGF in arsenic-treated cells in vitro (Figure 8b). We can indicate that after long-term arsenite exposure, matrine downregulated the production of VEGF by means of NOX2, which likely plays an anti-angiogenesis role in neoplasia, thus possibly providing a new intervention for tumor treatment.

## 3. Discussion

Abundant epidemiological evidence has shown a strong causal relationship between bladder cancer and long-term exposure to inorganic arsenic (iAs) in drinking water [35]. Chronic exposure to low concentrations of arsenite leads to cell transformation in several animal and cell models [36,37,38]. In vitro, we exposed SV-HUC-1 cells to 0.5 μM NaAsO_2_ for 40 weeks to establish a long-term arsenite-treated model. When arsenite is administered in the drinking water or diet at doses of 10 mg/L in mice and rats, preneoplastic proliferative bladder lesions appear [39]. In doses of 0, 1, 10, 25, 50, and 100 mg/L, cytotoxicity, cell proliferation, and hyperplasia of urothelial superficial cells increased in a dose-responsive manner, with maximum effects found at 50 mg/L in F344 rats for 5 weeks [40]. In our study, we selected 10 mg/L as the low-dose group and 50 mg/L as the high-dose group in vivo due to the long exposure time to arsenite. In both in vivo and in vitro experiments, we observed cell transformation, which included proliferation, migration, and angiogenesis in bladder epithelial cells.

Inflammation is a leading indicator of cancer development and progression [41]. The NLRP3 inflammasome was found to be activated in non-small-cell lung cancer, promoting the release of IL-1β and IL-18 [42]. Constitutive activation of the NLRP3 inflammasome causes late-stage human melanoma cells to spontaneously secrete IL-1β via Caspase 1 processing [43]. The potential role of inflammasomes in the early development of bladder cancer was suggested by the up-regulation of NLRP3 [44]. The NF-κB/NLRP3 signaling pathway can regulate TAM polarization to improve the tumor’s inflammatory microenvironment [45]. Arsenite induces a hippocampal inflammatory response and activates the NLRP3 inflammasome [46]. Our studies confirmed that arsenite can activate the NLRP3 inflammasome and promote the secretion of IL-1β and IL-18 in bladder epithelial cells. But how arsenite acts on NLRP3 is unclear.

ROS plays a role in the production of inflammatory cytokines and can activate the NLRP3 inflammasome [47]. Ros-mediated oxidative damage is a common denominator in arsenic pathogenesis, and exposure to arsenic enhanced the performance of ROS [48]. In SV-HUC-1 cells, arsenite increased the generation of ROS. NOX2 is the main enzyme for ROS production in the human body. We observed that the expression of NOX2 was also elevated in cells treated with chronic arsenite and in bladder epithelial cells of rats. Therefore, we deduced that arsenic may activate the NLRP3 inflammasome through ROS produced by NOX2.

A variety of natural products isolated from Chinese herbal medicines are widely used in cancer treatment due to their excellent antiproliferative, pro-apoptosis, anti-metastasis, autophagy, and immune regulation activities [24]. Studies have shown that bitter melon extract plays a cancer-suppressing role in breast cancer and neck squamous cell carcinoma (HNSCC). It inhibits tumor growth by regulating proliferation, autophagy, and immune function [49,50]. Polyphyllin II inhibits migration and invasion by regulating EMT-associated factors and MMPs in human bladder cancer [51]. Homogeneous Polyporus polysaccharide resets tumor-associated macrophages through NF-κB/NLRP3 signaling to inhibit bladder cancer [45].

Matrine has a positive protective effect against oxidative stress injury and inflammation in the murine heart [52]. Matrine inhibits the secretion of IL-1β via the MyD88/NF-κB pathway and the NLRP3 inflammasome in primary porcine alveolar macrophages [53]. We demonstrated that matrine has the ability to reduce the expression of NLRP3 as well as the secretion of IL-1β and IL-18 in bladder epithelial cells. These results provide further proof that matrine has excellent efficacy in downregulating inflammatory manifestations.

Given the established evidence that arsenic can induce the activation of the NLRP3 inflammasome via the production of ROS by NOX2, our study aims to investigate the potential impact of matrine on ROS and NOX2. Firstly, the levels of ROS were assessed in SV-HUC-1 cells. It was observed that the treatment with matrine resulted in a decrease in ROS levels. Additionally, the expression of NOX2 was found to be downregulated by matrine. A study has demonstrated that the presence of elevated levels of arsenic in drinking water is linked to an increased risk of cancer. However, it is important to note that exposures to lower levels of arsenic are generally not associated with such risks. The occurrence of a toxicological mode of action potentially linked to the result necessitates an adequate dosage to induce the elevated formation of tumors [54]. We selected a concentration of 50 mg/L, which represents a higher dose of arsenite, for the administration of matrine and apocynin (a NOX2 inhibitor) in our study. The findings in animal models align with our observations in cellular studies. Apocynin demonstrated inhibitory effects on the expression of NOX2, while matrine exhibited comparable efficacy in suppressing NOX2. It has been reported that the expression of NADPH oxidase subunits gp91phox and p47phox induced by Oaβ was markedly attenuated by the administration of matrine [28].

A decrease in the protein levels of Cyclin D1 and PCNA was observed following the treatment of matrine in vivo and long-term arsenite-treated cells. Under the administration of matrine, the acceleration of the cell cycle induced by arsenic was attenuated. Matrine exhibited a significant reduction in cell viability in arsenite-treated SV-HUC-1 cells, suggesting its potential as an inhibitor of arsenic-induced cell proliferation.

In the investigation of cell migration and angiogenesis, a significant impact was observed in the inhibition of arsenic-induced cell migration and angiogenesis. Considering that the expression of NOX2 might have an impact on VEGF [55], immunofluorescence was employed to investigate the expression and localization of VEGF in vivo. Therefore, matrine can arrest arsenic-induced bladder epithelial cell transformation. Now that matrine has been found to possess inhibitory effects on NOX2, it is possible that the process of NOX2 inhibition can be achieved through the use of matrine.

In summary, the findings of this study indicate that arsenite exposure resulted in an upregulation of NOX2 expression and facilitated the transformation of bladder epithelial cells. Matrine plays a role in cutting down NOX2 and alleviating the transformation of bladder epithelial cells. Our findings demonstrate that matrine has the potential to serve as a therapeutic intervention for the treatment of bladder cancer induced by arsenite exposure.

## 4. Materials and Methods

### 4.1. Cell Culture

The SV-40 immortalized human uroepithelial cells (SV-HUC-1) were purchased from the American Type Culture Collection (ATCC, Manassas, VI, USA). The SV-HUC-1 cells were routinely cultured with F-12K medium (Sigma, Livonia, MI, USA) containing 10% fetal bovine serum (Sigma, USA) and 1% penicillin-streptomycin solution (Biochrom, Berlin, Germany) and incubated in a humidified atmosphere with 5% CO_2_ at 37 °C. The NaAsO_2_ solution was prepared in sterile PBS. To ensure continuous exposure to NaAsO_2_, SV-HUC-1 cells were cultured in a medium containing 0 or 0.5 μM NaAsO_2_ for about 40 weeks (T40W) and were added fresh medium every day. Cells were passaged once every 3–4 days. To evaluate the effect of matrine (MAT, Aladdin, Shanghai, China), SV-HUC-1 cells were treated with matrine (2 mg/mL, diluted by F-12K medium) for 24 h. The dose of matrine was selected by the Cell-Counting Kit-8 assay. According to previous studies, 0.5 μM NaAsO_2_ was chosen as the exposure dose in long-term exposure experiments.

### 4.2. Reagents and Antibodies

Sodium arsenite (Sigma, Steinheim, Germany, purity > 99.0%) and the NaAsO_2_ solution were prepared in sterile PBS. To ensure the toxicity of NaAsO_2_, it was prepared immediately before every experiment. Apocynin (APO) was purchased from Abcam (Cambridge, MA, USA). Antibodies for E-cadherin and Vimentin were obtained from Cell Signaling Technology (Danvers, MA, USA). Cyclin D1, PCNA, NLRP3, Caspase 1, NOX2, GAPDH, and β-actin antibodies were purchased from Abclonal (Wuhan, China). Antibodies for VEGF, IL-1β, and IL-18 were purchased from Proteintech (Rosemont, IL, USA). The secondary antibodies were purchased from LICOR Biosciences (Lincoln, NE, USA).

### 4.3. Western Blot Analysis

Total protein was extracted by ice-cold RIPA buffer (Beyotime, Shanghai, China) supplemented with protease inhibitors (Sigma, Germany). Protein concentrations were determined by the Bicin-Choninic Acid (BCA) Protein Assay Kit (Beyotime, Shanghai, China). Each protein sample was separated by sodium dodecyl sulfate-polyacrylamide gel electrophoresis (SDS-PAGE) (Beyotime, Shanghai, China) and transferred onto the Polyvinylidene-Fluoride (PVDF) membrane (Millipore, Darmstadt, Germany). Membranes were blocked and incubated with primary antibodies overnight at 4 °C. The membranes were then washed with Phosphate-Buffered Saline with Tween-20 (PBST) buffer three times and incubated with acceptable secondary antibodies for 1 h at room temperature. Protein signals were detected by the LICOR Odyssey Infrared Imaging System (LI-COR Biosciences, Lincoln, NE, USA). The intensity of the signals was quantified using Image J 1.42q software tools.

### 4.4. RNA Extraction and Real-Time Quantitative PCR

Total RNA was extracted from cells with Trizol reagent (Takara, Dalian, China) and was reversely transcribed to cDNA using the PrimeScript^®^ RT reagent Kit with gDNA Eraser (Takara, Dalian, China). Real-time quantitative PCR (RT-qPCR) of cDNA was performed on the Quant Studio 6 Flex QRT-PCR system (Life Technologies, Carlsbad, CA, USA) using SYBR.

Premix EX Taq Kit (Takara, Dalian, China). The relative amount of mRNA normalized to *GAPDH* gene expressions was calculated using the ΔΔCT method. Specific forward and reverse primer sequences were shown in Table 1.

### 4.5. Cell Viability Analysis

Cells were seeded into 96-well plates at a density of 5 × 10^4^/mL and treated with matrine (2 mg/mL) for 24 h (Supplement Material). The viability of cells was evaluated by the Cell-Counting Kit-8 (CCK-8, Beyotime, Shanghai, China). A total of 10 μL of CCK-8 reagent was added to each well and incubated for 1 h in the dark. The absorbance of each well was read on a microplate reader (Labsystems, Vantaa, Finland) at 450 nm.

### 4.6. Wound Healing Analysis

Cells were seeded into 12-well plates and grown to 100% confluence. Confluent monolayer cells were scratched across the middle of the plate using a 200 μL sterilized micropipette tip and then gently washed with PBS three times to remove the suspended cells, then added to a Serum-free medium containing suitable reagents. The cells were cultured for 24 h at 37 °C. The “wound” area was captured at 0, 12, and 24 h after scratching by an inverted microscope (10× objective) (Leica, Wetzlar, Germany) and measured using Image J software tools.

### 4.7. Cell Cycle Assay

The cell cycle was analyzed using a Cell Cycle Analysis Kit (KeyGEN Biotech, Nanjing, China) according to the manufacturer’s instructions. Briefly, the cells were harvested after 24 h and fixed in ice-cold 70% ethanol overnight at −20 °C. After washing twice with ice-cold PBS, the cells were stained with 0.5 mL propidium iodide for 30 min in the presence of RNase A (20 μL), and then acquired in the BD flow cytometer (Becton, Dickinson, NJ, USA).

### 4.8. Reactive Oxygen Species Assay

ROS generation was detected according to the manufacturer’s instructions for the ROS Assay Kit (Beyotime, Shanghai, China) and the reference [56]. Briefly, cells were washed twice with PBS and incubated with 10 μM DCFH-DA at 37 °C for 30 min. Then cells were washed three times with Serum-free medium, twice with PBS, and analyzed by BD flow cytometer (Becton, Dickinson, NJ, USA).

### 4.9. Animals and Treatment

Wistar rats (weight of 100–120 g) were obtained from the Laboratory Animal Center of China Medical University and randomly divided into five groups (6 rats per group): control group, low arsenite-treated group (10 mg/L NaAsO_2_), high arsenite-treated group (50 mg/L NaAsO_2_) with three different groups. Control group rats were treated with distilled water for twelve weeks. Low arsenite-treated rats were supplied with distilled water containing 10 mg/L NaAsO_2_ for twelve weeks. High arsenite-treated rats were provided with distilled water containing 50 mg/L NaAsO_2_ for twelve weeks, and two of the three groups individually irrigated 20 mg/kg apocynin (APO) and 20 mg/kg matrine (MAT) once per day in the ninth week for three weeks. Rats in other groups received irrigation with the same amount of saline solution. All rats were provided a regular diet and water ad libitum and raised under a 12 h light/dark cycle with a constant temperature (24 °C) and relative humidity (45–55%). At the end of twelve weeks, the rats were fasted overnight and euthanized by intraperitoneal injection of 20% rartan solution (0.7 mL/100 g body weight), and the bladders were removed and fixed in 4% paraformaldehyde for 48 h. The fixed tissues were embedded in paraffin and cut into 5 μm-thick sections for further experiments. All experimental protocols were approved by the Ethics Review Committee for Animal Experimentation of the China Medical University (SYXK 2018-0008, Shenyang, China).

### 4.10. Immunohistochemistry

The method of immunohistochemistry refers to the references of previous studies in our group [12]. Sections were deparaffinized with xylene and rehydrated successively in ethanol. After treating with 0.01 M citrate buffer for antigen retrieval, endogenous peroxidase was inactivated with 3% H_2_O_2_ for 10 min, then blocked with 5% BSA for half an hour at 37 °C and incubated with primary antibodies overnight at 4 °C. Primary antibodies were used as follows: NLRP3 (1:200; Abclonal, Wuhan, China), IL-1β (1:200; Abclonal, Wuhan, China), E-cadherin (1:200; Proteintech, USA), Vimentin (1:100; Sangon, Shanghai, China), and Cyclin D1 (1:500; Abclonal, Wuhan, China), and covered with goat anti-rabbit secondary antibody at 37 °C for 30 min. Sections were rinsed again in PBS and incubated in 100 μL SABC (BOSTER, Wuhan, China) for 30 min. After that, the sections were colored by 3,3′-Diaminobenzidine (DAB) (BOSTER, Wuhan, China) and counterstained with hematoxylin. The slides were observed and captured using a light microscope (Leica, Germany), and the integrated optical density average (IOD) of four randomly selected fields was analyzed by ImagePro Plus 6.0 software (Media Cybernetics, Inc., Rockville, MD, USA).

### 4.11. Immunofluorescence

For immunofluorescence, sections were deparaffinized with xylene and rehydrated successively in ethanol. After treatment with 0.01 M citrate buffer for antigen retrieval, the slides were blocked with Immunol Staining Blocking Buffer (Beyotime, Shanghai, China) for 30 min at 37 °C, and incubated overnight with appropriate primary antibodies at 4 °C. Primary antibodies were used as follows: VEGF (1:200; Abclonal, Wuhan, China) and NOX2 (1:200; Proteintech, USA). The sections were then washed with PBS buffer three times and incubated with an Alexa Fluor 555-conjugated secondary antibody (Invitrogen, Thermo, Waltham, MA, USA) for 2 h at 4 °C. Washed with PBS buffer three times, slides were counterstained with 4′,6-diamidino-2-phenylindole (DAPI) (BOSTER, Wuhan, China) for 10 min at room temperature to counter the nuclei. All images were observed and obtained by a fluorescence microscope (Nikon, Tokyo, Japan), and the lens number of microscopy is MRP70200. The immunofluorescence densities of four randomly selected fields were analyzed by Image J software tools.

### 4.12. Statistical Analysis

All the experiments were performed at least three times. The data were expressed as the mean standard deviation (SD). Differences between the groups were analyzed by an Independent-Samples *t*-test or one-way ANOVA using SPSS 27.0 software. If the difference between groups is statistically significant, the Least-Significant Difference (LSD) is used for pairwise comparison. Statistical significance was considered to be a two-sided *p* value < 0.05.

## 5. Conclusions

Chronic arsenic exposure induced changes in bladder epithelial cells, which gave them tumor cell characteristics and increased the expression of NLRP3 inflammation-related factors, ROS and NOX2. Matrine can inhibit the malignant transformation of bladder epithelial cells caused by chronic arsenic exposure through the NOX2 pathway. Matrine is a naturally occurring alkaloid that was extracted from Sophora radix. It has good potential for drug development and application in the treatment of bladder cancer brought on by endemic arsenic poisoning.

## Figures and Tables

**Table 1:** PCR primer sequence.

Gene	Forward Primer (5′-3′)	Reverse Primer (5′-3′)
*Cyclin D1*	GCTGCGAAGTGGAAACCATC	CCTCCTTCTGCACACATTTGAA
*PCNA*	GGCCGAAGATAACGCGGATAC	GGCATATACGTGCAAATTCACCAG
*E–cadherin*	GCTGGACCGAGAGAGTTTCC	CAAAATCCAAGCCCGTGGTG
*Vimentin*	ACCAGCTAACCAACGACAAAG	AAAGATTGCAGGGTGTTTTCG
*VEGF*	CCGCAGACGTGTAAATGTTCCT	TTCCGGTGAGAGGTCTGGTTC
*NLRP3*	GCGTGGTCTTGAATTCCTCA	GGCACACGGATGAGTCTTT
*IL–1β*	GCCAGTGAAATGATGGCTTATT	AGGAGCACTTCATCTGTTTAGG
*IL–18*	GCTGAAGATGATGAAAACCTGG	CAAATAGAGGCCGATTTCCTTG
*Caspase 1*	CATCCCACAATGGGCTCTGT	GCATCTGCGCTCTACCATCT
*NOX2*	AAGATGCGTGGAAACTACCTAA	TTTTTGAGCTTCAGATTGGTGG
*GAPDH*	TGTTGCCATCAATGACCCCTT	CTCCACGACGTACTCAGCG

## Data Availability

The raw data supporting the conclusions of this article will be made available by the authors upon request.

## Supplementary Materials

The following supporting information can be downloaded at: https://www.mdpi.com/article/10.3390/ijms25168878/s1.
