# Supplementary material for: Matrine Suppresses Arsenic-Induced Malignant Transformation of SV-HUC-1 Cells via NOX2
Source: Int J Mol Sci. 2024 Aug 15;25(16):8878. doi: 10.3390/ijms25168878 (PMC11354282; doi:10.3390/ijms25168878)
Supplement: Supplementary file 1 [file ijms-25-08878-s001.zip › ijms-3135807-supplementary.pdf]

Supplementary Material

1. Supplementary Figures

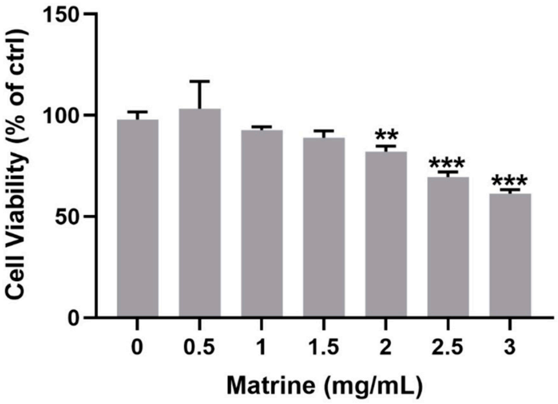

Figure S1. Effect of matrine on cell viability. SV-HUC-1 cells were treated with different concentrations of matrine solution for 24 h. SV-HUC-1 cell viability was significantly reduced to 82.07% after the treatment of 2 mg/mL matrine ($P < 0.05$). In the 2.5 and 3 mg/mL matrine groups, the viability of SV-HUC-1 cells decreased to 69.52% and 61.36%, respectively. Therefore, a 2 mg/mL matrine solution was chosen for subsequent experiments Dosage of medication. Data were presented as mean ± SD. * $P < 0.05$, ** $P < 0.01$, and *** $P < 0.001$ compared to the 0 mg/mL matrine group (n = 3).
